# Cost-Effectiveness of Adding Bedaquiline to Drug Regimens for the Treatment of Multidrug-Resistant Tuberculosis in the UK

**DOI:** 10.1371/journal.pone.0120763

**Published:** 2015-03-20

**Authors:** Lara J. Wolfson, Anna Walker, Robert Hettle, Xiaoyan Lu, Chrispin Kambili, Andrew Murungi, Gerhart Knerer

**Affiliations:** 1 Janssen Pharmaceutica NV, Beerse, Belgium; 2 HERON Commercialization, London, United Kingdom; 3 Janssen Global Services LLC, Raritan, New Jersey, United States of America; 4 Janssen (Janssen-Cilag), High Wycombe, United Kingdom; University Hospital Schleswig Holstein, GERMANY

## Abstract

**Objective:**

To evaluate the cost-effectiveness of adding bedaquiline to a background regimen (BR) of drugs for multidrug-resistant tuberculosis (MDR-TB) in the United Kingdom (UK).

**Methods:**

A cohort-based Markov model was developed to estimate the incremental cost-effectiveness ratio of bedaquiline plus BR (BBR) versus BR alone (BR) in the treatment of MDR-TB, over a 10-year time horizon. A National Health Service (NHS) and personal social services perspective was considered. Cost-effectiveness was evaluated in terms of Quality-Adjusted Life Years (QALYs) and Disability-Adjusted Life Years (DALYs). Data were sourced from a phase II, placebo-controlled trial, NHS reference costs, and the literature; the US list price of bedaquiline was used and converted to pounds (£18,800). Costs and effectiveness were discounted at a rate of 3.5% per annum. Probabilistic and deterministic sensitivity analysis was conducted.

**Results:**

The total discounted cost per patient (pp) on BBR was £106,487, compared with £117,922 for BR. The total discounted QALYs pp were 5.16 for BBR and 4.01 for BR. The addition of bedaquiline to a BR resulted in a cost-saving of £11,434 and an additional 1.14 QALYs pp over a 10-year period, and is therefore considered to be the dominant (less costly and more effective) strategy over BR. BBR remained dominant in the majority of sensitivity analyses, with a 81% probability of being dominant versus BR in the probabilistic analysis.

**Conclusions:**

In the UK, bedaquiline is likely to be cost-effective and cost-saving, compared with the current MDR-TB standard of care under a range of scenarios. Cost-savings over a 10-year period were realized from reductions in length of hospitalization, which offset the bedaquiline drug costs. The cost-benefit conclusions held after several sensitivity analyses, thus validating assumptions made, and suggesting that the results would hold even if the actual price of bedaquiline in the UK were higher than in the US.

## Introduction

Multidrug-resistant tuberculosis (MDR-TB), a disease caused by strains of TB that are resistant to the two most important first-line anti-TB drugs, isoniazid and rifampicin, is a growing public health problem [1]. In the UK, the incidence of drug-susceptible TB (DS-TB) has held steady at approximately 9,000 cases per year over the last decade, while a gradual but considerable increase in the proportion of drug-resistant TB has been observed [2]. In addition, recent years have seen annual growth in the number and rate of incident cases of extensively drug-resistant TB (XDR-TB) and totally drug-resistant TB (TDR-TB) [2]. Similar trends have been observed in other high-income countries, including France, Spain, and Sweden [3]. Consequently, the adequate and appropriate treatment of MDR-TB is of growing public health concern, and improved treatment strategies may be required to treat complex cases of MDR-TB.

MDR-TB is difficult to treat, typically requiring a regimen of toxic and poorly tolerated second-line anti-TB drugs, including injectable drugs, for a period of 18 to 24 months [4–6]. Poor efficacy of the second-line agents and erratic access to these drugs, coupled with medication-related adverse effects and increased likelihood of decreased adherence to drugs during the lengthy treatment period, all lead to poor outcomes in the treatment of MDR-TB; the WHO reports that favorable treatment outcomes are achieved in only 48% of MDR-TB cases globally [7]. There is therefore a clear need for new treatments that improve treatment outcomes in a higher proportion of patients with MDR-TB [8,9].

Between December 2012 and May 2014, the European Medicines Agency (European Union [EU]), the Food and Drug Administration (United States [US]), Russian regulatory authority, and the South Korean Ministry of Food and Drug Safety, have granted marketing authorization to bedaquiline, the first novel treatment to be licensed for use in MDR-TB in over 40 years [10,11]. In the EU and the US, bedaquiline is approved for use as part of an appropriate combination therapy in adult patients with pulmonary MDR-TB, when an effective treatment regimen cannot otherwise be provided for reasons of resistance or tolerability. Bedaquiline should be administered over a 24-week period, ideally using directly observed therapy (DOT)[12]. A randomized clinical trial has shown that the addition of bedaquiline to a background treatment regimen (BR) for 24 weeks resulted in significantly shorter time to sputum culture conversion (p<0.0001) and a significantly higher proportion of subjects with culture conversion (p = 0.008) compared to BR alone [13–15].

It is intuitive that the addition of bedaquiline to a standard MDR-TB drug regimen would add to drug costs. However, it is important to assess the overall economic impact of such incremental costs. The cost-effectiveness of bedaquiline as an add-on treatment in MDR-TB was recently assessed by the World Health Organization (WHO), who commissioned an analysis assessing the use of bedaquiline from the perspective of TB programs in a range of six low- and middle-income countries (Russia, Estonia, Philippines, Peru, Nepal and China) [16]. The analysis considered the costs and effectiveness of two strategies: bedaquiline plus BR, and BR alone over a period of 20 months, using outcomes from published literature and initial results from the bedaquiline clinical trial program. The WHO analysis concluded that bedaquiline is highly likely to be cost-effective in most environments, with cost-savings in environments where treatment and management costs for MDR-TB are high [16].

In high income countries, such as the UK, patients with MDR-TB are almost always hospitalized, typically confined to a negative pressure isolation room to reduce the risk of disease transmission. Inpatient care is provided until sputum culture conversion is achieved, often resulting in lengthy periods of hospital stay, and incurring significant costs to the health care system. A UK study estimated that the mean cost of managing a single case of pulmonary MDR-TB was in excess of £60,000 (year 2000 value) [17], while the cost of MDR-TB in the EU has recently been estimated at €57,213 per case [18]. No cost-effectiveness analysis has been conducted on the use of bedaquiline in high income countries; as such, there is no information to inform whether the introduction of bedaquiline would be an effective use of healthcare resources in such countries. In the EU, bedaquiline is designated as an orphan medicinal product (defined as a disease with incidence of less than 5 per 10,000 of the population [19,20]). Therefore, when making economic assessments in orphan diseases, Health Technology Appraisal organizations such as NICE in the UK may consider equity weighting to modify the Quality Adjusted Life Year (QALY) gains, increasing the usual willingness-to-pay threshold from £20,000-£30,000 [21], to as high as £50,000 [22].

The objective of this study was to estimate the cost-effectiveness of adding bedaquiline to a BR of at least three proven anti-TB drugs compared with BR alone, in the treatment of MDR-TB in a high income setting, using the UK as an example.

## Methods

### Summary of evaluation

A cohort-based Markov state transition model [23] was developed to evaluate the long-term costs, and effectiveness of adding bedaquiline to a BR, compared with BR alone, in the treatment of adult patients with pulmonary MDR-TB. The characteristics of the cohort evaluated in simulation were based on the enrolled population in C208 Stage 2, a phase II, placebo-controlled randomized trial of bedaquiline in newly diagnosed MDR-TB patients [13], which is the primary source of efficacy data in the simulation.

The simulation base case was conducted from the perspective of the UK National Health Service (NHS) and Personal Social Services (PSS). The effectiveness of treatment was evaluated in terms of QALYs, the recommended measure of effectiveness in economic evaluations for health technology appraisals in the UK [24], and the Disability Adjusted Life Year (DALY), which is typically used in evaluating effectiveness for neglected diseases in the developing world [25]. Both costs and effectiveness were discounted in the base case at an annual rate of 3.5% per annum [26], with varying discount rates explored in sensitivity analyses. A half cycle correction was applied to estimate costs and outcomes.

The model simulated costs and effectiveness over a time horizon of 10 years in order to capture the major future consequences of treatment with bedaquiline. The primary outcome of the analysis was the incremental cost per QALY gained, calculated by dividing the incremental cost with the incremental QALYs gained when comparing bedaquiline plus BR with BR alone. Secondary outcomes included the total and per-patient costs, QALYs, DALYs, and the incremental cost per DALY avoided. Total costs and total effects for treatment were reported for a cohort of 20 patients with MDR-TB, representing the estimated number of patients with MDR-TB who would likely be eligible for treatment with bedaquiline in the UK in 2014 [14].

### Summary of model structure

**Fig 1:**
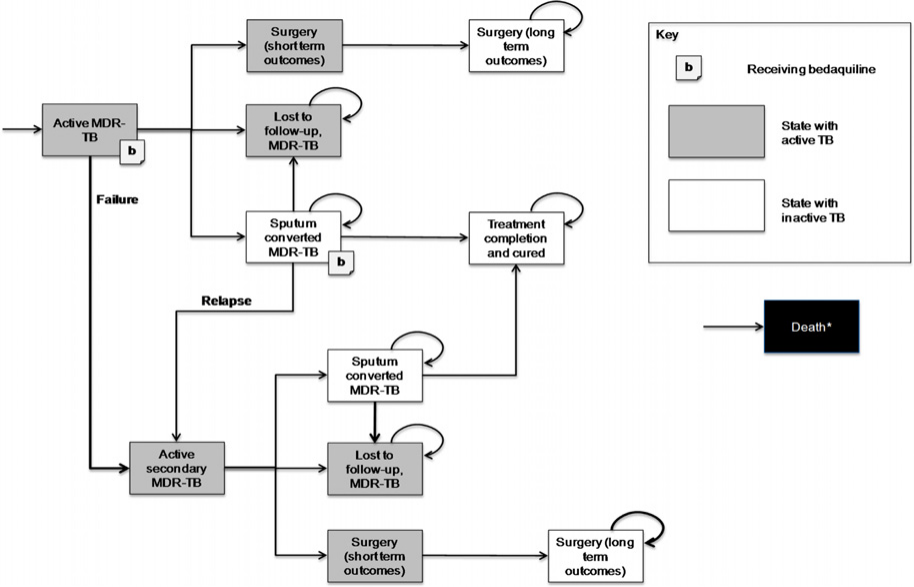
Markov model state structure. MDR-TB: multi-drug resistant tuberculosis; TB: tuberculosis. *Transitions to the death state are possible from every state, but not shown on the diagram for clarity.

A *de novo* model structure was developed to simulate the clinical pathway and the subsequent long-term outcomes of treatment in patients with newly diagnosed MDR-TB (Fig. 1). The model structure consisted of six core health states, corresponding to states of sputum culture positivity to MDR-TB (‘active MDR-TB’ and ‘Active secondary / Relapse MDR-TB’), sputum culture converted MDR-TB, surgery (‘short-term outcomes’ and ‘long-term outcomes’), lost to follow-up, treatment completion and death. The simulated cohort transited between health states over a fixed 28-day cycle period.

The goal of drug treatment in the simulation was to induce sputum culture conversion (transition to sputum culture converted MDR-TB) in the patient cohort, and maintain converted state until treatment completion and assumed cure of MDR-TB (transition to treatment completion). In the base case, treatment completion was achieved if patients did not fail treatment in the 12 months following sputum culture conversion, a definition that was assumed to align with UK and WHO guidelines [4,27]. Failure to complete treatment in the simulation was due to the reappearance (reversion) of sputum positive MDR-TB (transition to ‘active secondary MDR-TB’), loss to follow-up, or death. In the model, tunnel states were used to track the time since initial culture conversion, with the risk of reversion, loss to follow-up, and death applied to the cohort for each cycle in which they occupied the tunnel states.

In the simulation base case, treatment completion was assumed equivalent to the cure of TB as defined by the WHO (five consecutive negative cultures from samples collected at least 30 days apart in the final 12 months of treatment) [28]. Subsequently, the cohort who occupied this state was assumed to have the same health characteristics (quality of life, risk of mortality) as the UK general population. Once cured, this cohort was assumed to be at zero risk of re-developing or being re-infected with MDR-TB during the simulation period. This conservative assumption was tested in the sensitivity analysis.

In the simulation, patients who failed to achieve culture conversion during the first year of the simulation were considered treatment failures, and automatically transited to the ‘active secondary MDR-TB’ state at month 12 to begin a new treatment program. The goal of the new drug treatment program was to accelerate and maintain sputum culture conversion. Treatment completion was modeled in the same manner as for first treatment, with failure to complete treatment due to reversion, loss to follow-up or death. In the base case, the likelihood of culture conversion was lower in this group to reflect the refractory nature of secondary (relapse) MDR-TB. In the simulation, patients who failed to achieve culture conversion with the new program were assumed to occupy the active secondary MDR-TB state until death, loss to follow-up or surgical intervention. These patients were considered to have chronic TB.

Surgical intervention was modeled using two health states to reflect the short-term (cost and risk of mortality for the surgery itself) and long-term (outcomes in patients with surgery) consequences of surgery. Information on the transition probabilities applied in the model can be found in Table 1.

**Table 1:** Transition probabilities.

Parameter	Value per 28-days (SE)	Source
Sputum culture conversion on BR, 0–8 weeks (log-normal)		
Scale parameter	4.99 (0.21)	Placebo-controlled phase II clinical trial C208 [13]
Shape parameter	0.73 (0.11)	Placebo-controlled phase II clinical trial C208 [13]
Sputum culture conversion on BR, 8–24 weeks (log-normal)		
Scale parameter	5.68 (0.40)	Placebo-controlled phase II clinical trial C208 [13]
Shape parameter	1.90 (0.27)	Placebo-controlled phase II clinical trial C208 [13]
Sputum culture conversion on BR, 24 + weeks (log-normal)		
Scale parameter	8.28 (1.09)	Placebo-controlled phase II clinical trial C208 [13]
Shape parameter	2.70 (0.82)	Placebo-controlled phase II clinical trial C208 [13]
Subsequent MDR		
Hazard ratio of subsequent MDR-TB (vs. initial MDR-TB)	0.54 (0.17)	Open-label, phase II clinical trial C209 [13]
Other events		
Probability of loss to follow-up	0.39% (0.0016)	[2]
Probability of surgery (<24 weeks)*	0% (0)	Placebo-controlled phase II clinical trial C208 [13]
Probability of surgery (>24 weeks)	0.38% (0.026)	Placebo-controlled phase II clinical trial C208 [13]
Probability of reversion	0.98% (0.0035)	Placebo-controlled phase II clinical trial C208 [13]
Probability of death	MDR-TB, no cure: 2.21%	[31] [30]
	MDR-TB, cured: 0.32%	[31] [30]
	XDR-TB, no cure: 2.69%	[31] [30]
	XDR-TB, cured: 0.39%	[31] [30]
Treatment effect		
Hazard ratio of bedaquiline (sputum culture conversion)	2.44 (0.57; 95% CI: 1.57–3.80)	Placebo-controlled phase II clinical trial C208 [13]
Hazard ratio of bedaquiline (relapse)	0.32 (0.25; 95% CI: 0.086–1.069)	Placebo-controlled phase II clinical trial C208 [13]

MDR-TB: multi-drug resistant tuberculosis; TB: tuberculosis; XDR-TB: extensively-drug resistant tuberculosis; SE: standard error; MDR: Multi-drug resistance

### Clinical data in the model

Data on the efficacy of treatment and the risk of morbidity and mortality in patients with MDR-TB was obtained from various sources, including C208 Stage 2, the phase II, placebo-controlled randomized trial of bedaquiline in newly diagnosed MDR-TB patients [13,15], and published literature [29–31].

Data used to directly model differences in the efficacy and safety of bedaquiline plus BR and BR alone were obtained from the randomized trial. The probability of surgical intervention, loss to follow-up, and death were based on the literature [2,13,29–31], with the same probabilities applied in the bedaquiline plus BR and BR alone simulations. Given the decreasing trend of surgical interventions observed in C208 Stage 2, this is a conservative approach. A summary of the literature data is provided in S1 Table.

In the simulation base case, bedaquiline plus BR was associated with an increased rate (and probability) of sputum culture conversion and reduced rate (and probability) of relapse or reversion compared with BR alone.

Sputum culture conversion rates in patients receiving BR alone were modeled by fitting parametric survival curves to patient-level data on the time-to-culture conversion in the placebo group of the phase II study. For the analysis, the trial horizon was segmented into three time periods (<8 weeks, 8 to 24 weeks, and >24 weeks), and survival curves were fitted to data observed during each period. The three curves provided a superior fit to the observed trend in sputum culture conversion in the study than a single curve fitted to the complete trial horizon. Standard survival distributions (e.g. exponential, Weibull) were explored and fitted to the data, and the log-normal distribution was chosen based on statistical model fit, assessed by Akaike’s information criterion (AIC) and Bayesian information criterion (BIC). In the model, the rate of sputum culture conversion was assumed to vary over time.

Sputum culture conversion rates in patients receiving bedaquiline plus BR were modeled using data from the trial, by multiplying the rate of culture conversion from the placebo group by the hazard ratio (or rate ratio) of culture conversion for bedaquiline versus placebo (Hazard ratio = 2.44, standard error = 0.57, 95% CI = 1.57–3.80).

The hazard ratio of culture conversion was applied for the duration of bedaquiline treatment (6 months), after which the rate of sputum culture conversion was the same as observed in the placebo group.

An additional benefit was modeled in terms of a lower rate of reversion in the bedaquiline treatment group compared to placebo. This was estimated by fitting a complementary log-log model to aggregated data of patients pooled from both the bedaquiline and placebo arms who experienced a reversion between weeks 24 and 120 of the phase II study, using the statistical software Winbugs [32]. The estimated hazard ratio of reversion following culture conversion for bedaquiline versus placebo was 0.32 (standard error = 0.25, 95% CI = 0.086–1.069). The reduced rate of reversion post-culture conversion was observed throughout the study and after cessation of bedaquiline. As such, the hazard ratio of reduced reversion post-conversion was applied for the duration of the simulation. This assumption was tested in the sensitivity analysis.

### Costs

Costs included in the model comprised direct medical costs, including drug acquisition costs, treatment monitoring costs, administered care (inpatient and outpatient) costs, and the cost of surgical intervention, where applicable. Key cost data inputs used in the model are summarized in Table 2.

**Table 2:** Costs and outcomes.

Parameter	Value per event / 28-days (SE)	Source
Cost of drug treatment		
Cost of bedaquiline (per 6-month course)	£18,800	Assumption—estimated from the publically available US price converted to Great British Pounds using common currency conversion, as the UK cost was not available at the time of the analysis
Cost of BR (intensive)	£692.70	Placebo-controlled phase II clinical trial C208 [13], [33]
Cost of BR (continuation)	£273.30	Placebo-controlled phase II clinical trial C208 [13], [33]
Cost of monitoring		
Bedaquiline + BR	Intensive phase: £62.37	Calculated based on resource use assumptions
	Continuation phase: £31.92	Calculated based on resource use assumptions
BR only	Intensive phase: £124.91	Calculated based on resource use assumptions
	Continuation phase: £94.46	Calculated based on resource use assumptions
Cost of hospitalization (negative pressure room)	£18,800 per initial stay (48 days)	[34], inflated to 2013 values
	£274.75 per excess bed day	[34], inflated to 2013 values
Cost of outpatient care	£1,882.27	Calculated based on resource use assumptions
Cost of surgical procedure	£6,706.88 (3,559)	[34], inflated to 2013 values
Successful surgery:	No additional cost	Assumption
Unsuccessful surgery:	£94.46	Same as continuation BR

BR: background regimen; SE: standard error; TB: tuberculosis

Unit costs for each drug in the BR, the cost of each monitoring resource, outpatient visits, and inpatient costs were sourced from publically available tariffs and formularies in the UK, and were inflated to 2013 values where necessary [27,33–35]. In the base case, the cost of bedaquiline in the UK was assumed to be the same as the US list price converted to Great British Pounds (GBP) using common currency conversion, as the UK cost had not yet been determined at the time of the analysis and the US price was the only publicly listed price available at the time of analysis. The impact of bedaquiline cost on simulation results was tested in sensitivity analyses, including determining the maximum price at which bedaquiline would be considered cost-effective.

Resource use data was obtained from various sources including an interview with a UK clinician who had over 10 years’ experience, as well as expert opinion from one of the co-authors (CK) with experience of treating MDR-TB patients. All costs were estimated for the year 2013, and are reported in GBP.

Drug acquisition and treatment monitoring costs varied between the intensive phase (the first 8 months) and the continuation phase of treatment, in line with UK and WHO guidelines for treatment programs in MDR-TB [27,33]. Treatment monitoring included blood tests, X-rays, audiometric testing (intensive phase only), kidney and liver function monitoring, and cardiac monitoring for QT prolongation (only for those receiving bedaquiline).

In the simulation base case, the cohort that occupied the active sputum MDR-TB state accrued hospital care costs up to month 18 of the simulation (as is the standard of care in the UK), after which they were assumed to be transferred to an outpatient setting. The cohort that occupied the active secondary MDR-TB state received up to 6 months of continuous hospital care, after which they also received ongoing outpatient care. The cohort that occupied the sputum converted states was assumed to receive outpatient monitoring and care until successful treatment completion. Although WHO guidelines recommend ambulatory care, hospital-based care remains the recommended standard of care in high income settings such as the UK [35], due to concerns over infectiousness and treatment adherence.

The cohort that occupied the short-term surgery state accrued the one-off cost of surgery. Patients who had an unsuccessful outcome following surgery incurred subsequent additional treatment costs. We did not directly account for any specific complications of surgery.

No costs were applied to cohorts that occupied the lost to follow-up, successful surgery, treatment completion or death states of the model.

### Health utilities and Disability weights

Utility weights (QALY), disability weights (DALY) and years of life lost (DALY) were sourced from the published literature, and used to estimate the effectiveness of treatment in the simulation. A summary of data is provided in S1 Table.

Utility weights were obtained from two sources; a UK study of dedicated outreach services for patients with TB in the UK, which reported in terms of active TB and inactive TB but receiving drug treatment, and a UK study of quality of life in the general population [36,37]. The utility weights were elicited using the EQ-5D questionnaire, with responses mapped to utility weights using the York tariff. Although it can be questioned as to whether or not utility weights derived for general TB patients would be reflective of the lower quality of life that may be experienced by MDR-TB patients, in the absence of such information these were thought to be the most suitable proxy data available.

The cohorts that occupied the active MDR-TB and lost to follow-up states were assigned the utility weight for active TB. The cohort who occupied the treatment completion state was assigned a utility weight estimated from the age and gender-matched general population in the UK (EQ-5D data). The cohort who occupied the sputum converted MDR-TB states were assigned a utility weight that was dependent on the time since conversion, with a longer duration of sustained conversion being associated with an improvement in utility weight up to the utility weight for the general population (treatment completion). As such, the utility weight for sputum converted MDR-TB was estimated by linear interpolation of the weights for active TB (lower bound) and the general population (upper bound).

Disability weights and years of life lost due to premature mortality were sourced from two sources: a global burden of disease study [38], which reported disability weights for active TB, and the UK life tables from the office for national statistics [39]. The disability weight for treatment completion was assumed to be zero (i.e. no disability). The same assumptions were applied for the DALY calculation as used for the QALY calculation.

### Sensitivity analysis

Deterministic sensitivity analyses were conducted to assess the sensitivity of model results to alternative clinical assumptions and variations in input data. This included analysis of the impact of alternative structural assumptions on simulation results, including a shorter time horizon of two years, and the assumptions that patients may re-develop TB following treatment completion (reoccurrence), may acquire additional drug resistance, and do not experience a reduction in the rate of sputum culture reversion after having initially converted following treatment with bedaquiline. Additional analyses were conducted to test the impact of variations in data inputs on simulation results, including the proportion of patients receiving inpatient care, the rate of sputum culture conversion in the BR alone group, and the cost of bedaquiline.

A probabilistic sensitivity analysis was also performed to estimate the joint parametric uncertainty surrounding the incremental costs, incremental effectiveness, and incremental cost-effectiveness ratio of bedaquiline plus BR versus BR alone. The probabilistic analysis provided an estimate of the probability that bedaquiline plus BR is considered cost-effective versus BR alone at various thresholds of affordability (willingness to pay). Probabilistic distributions and parameters for this analysis can be found in S2 Table.

## Results

### Results of the base case

Over the 10-year time horizon, the total discounted cost and discounted QALYs for the single cohort of 20 patients assigned to bedaquiline plus BR was £2,170,394 and 105.09 QALYs, using the US list price for bedaquiline of £18,800 (Table 3). In comparison, the total discounted cost and discounted QALYs for BR alone was £2,403,442 and 81.80 QALYs. The treatment strategy of bedaquiline plus BR versus BR alone was therefore less costly (-£233,048) and more effective (23.28) than BR alone.

**Table 3:** Total and incremental results for bedaquiline + BR vs. BR alone (UK base case).

	Bedaquiline + BR	BR only	Incremental (Bedaquiline + BR vs. BR)
Total Costs	£2,170,394	£2,403,442	-£233,048
Per patient cost	£106,487	£117,922	-£11,434
Total QALYs gained	105.09	81.80	23.28
Per patient QALYs	5.16	4.01	1.14
Total DALYs lost	187.80	280.83	-93.04
Per patient DALYs lost	9.21	13.78	-4.56
Incremental cost per QALY gained	Dominates (-£10,008.75)		
Incremental cost per DALY avoided	Dominates (-£2,504.95)		

All costs reported in 2013 values

BR: background regimen; QALYs: quality adjusted life-years; DALYs: disability adjusted life-years

On an individual patient basis, the average total discounted costs and QALYs were £106,487 and 5.16, and £117,922 and 4.01, for bedaquiline plus BR and BR alone respectively. Thus, for each patient treated with bedaquiline, the average cost-saving versus standard of care (BR alone) was £11,434 over a 10-year time period.

The DALY burden for treatment with bedaquiline was 187.80 DALYs compared with 280.83 DALYs when using BR alone for a cohort of 20 patients (Table 3). This equates to a per patient DALY burden of 9.21 in patients receiving bedaquiline plus BR versus 13.78 in patients receiving BR alone. The dominant contributor to DALYs was the years of life lost, with 8.81 and 13.21 discounted years of life lost for bedaquiline plus BR and BR alone, respectively. The incremental DALYs avoided with bedaquiline plus BR versus BR alone was 93.04 DALYs for the cohort of 20, and 4.56 DALYs for the average individual patient.

The total costs of treatment and per patient costs, split by category, are presented in Table 4. The total cost of treatment with bedaquiline plus BR (£2,170,394) comprises principally hospitalization costs (£1,091,146, 50%), anti-TB drug costs (£529,904, 24%), and outpatient costs (£478,291, 22%). Similarly, the cost of BR alone (£2,403,442) comprises hospitalization costs (£1,639,622, 68%), outpatient costs (£516,572, 22%) and anti-TB drug costs (£192,433, 8%).

**Table 4:** Total and per patient costs, split by category (UK base case).

Cost category	Bedaquiline + BR (total)	Bedaquiline + BR (per patient)	BR only (total)	BR only (per patient)
Hospitalization costs	£1,091,146 [50%]	£53,536 [50%]	£1,639,622 [68%]	£80,446 [68%]
Outpatient care	£478,291 [22%]	£23,467 [22%]	£516,572 [22%]	£25,345 [22%]
Anti-TB drugs	£529,904 [24%]	£25,999 [24%]	£192,433 [8%]	£9,441 [8%]
Monitoring costs	£40,778 [2%]	£2,001 [2%]	£19,718 [1%]	£967 [1%]
Surgical costs	£5,093 [<1%]	£250 [<1%]	£9,914 [<1%]	£486 [<1%]
Contact tracing costs	£25,182 [1%]	£1,236 [1%]	£25,182 [1%]	£1,236 [1%]
Total costs	£2,170,394 [100%]	£106,487 [100%]	£2,403,442 [100%]	£117,922 [100%]

All costs reported in 2013 values

BR: background regimen; TB: tuberculosis

### Results of sensitivity analysis

**Fig 2:**
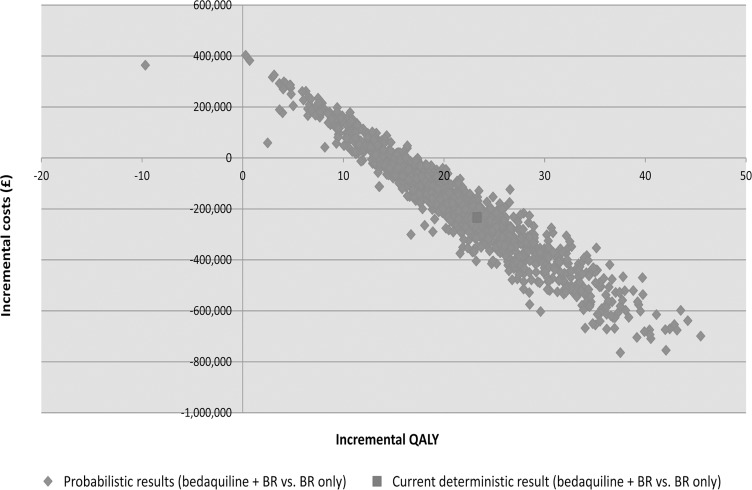
Cost-effectiveness plane for bedaquiline + BR versus BR only from a UK NHS and PSS perspective. BR: background regimen; NHS: National Health Service; PSS: Personal Social Services; QALY: quality adjusted life-year

**Fig 3:**
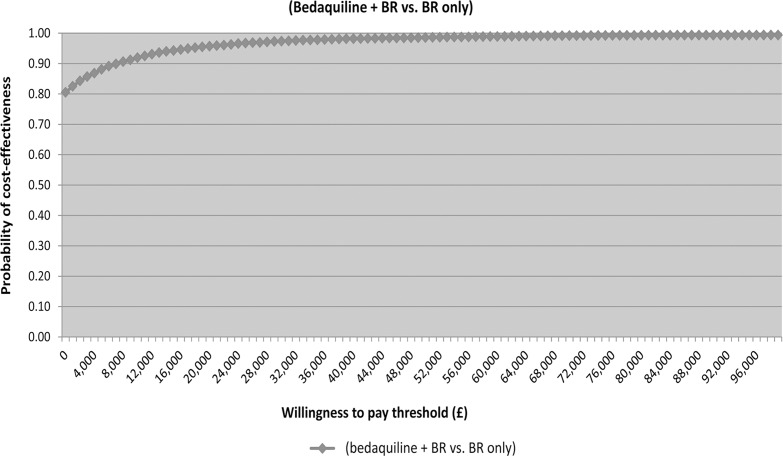
Cost-effectiveness acceptability curve for bedaquiline + BR versus BR only from a UK payer perspective (assuming price of £18,800 per treatment course). BR: background regimen

The results of the probabilistic sensitivity analysis are presented in Fig. 2 and Fig. 3. The probability that bedaquiline plus BR is cost-effective versus BR alone at an affordability threshold of £20,000 per QALY gained [24] and £50,000 per QALY gained [22] was 96% and 99%, respectively. The strategy of bedaquiline plus BR was cost-saving (and dominant) versus BR alone in 81% of probabilistic simulations.

**Fig 4:**
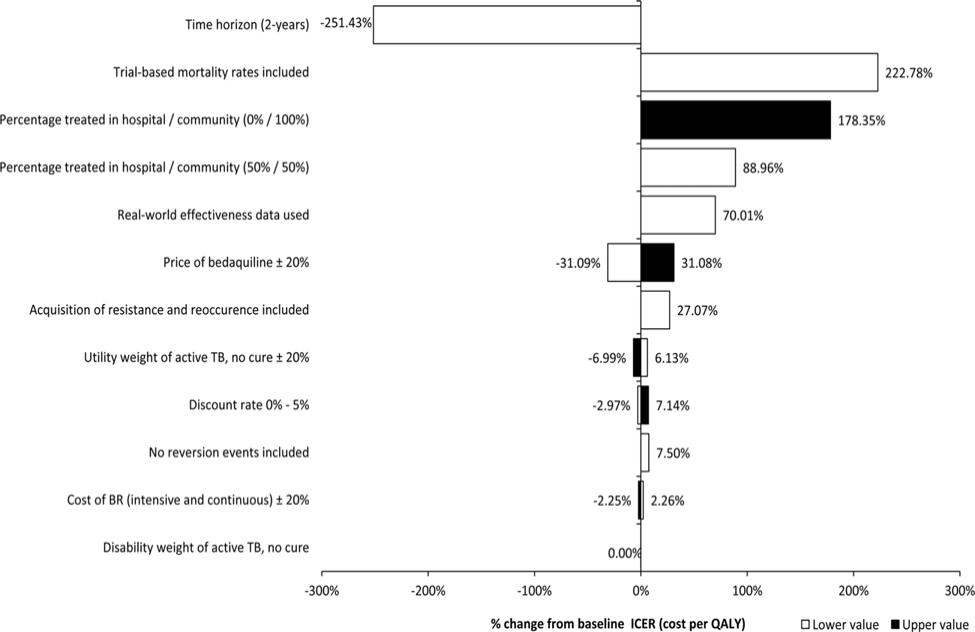
Tornado diagram representing deterministic sensitivity analysis based on incremental cost per QALY. * Fixed ranges of +/- 20% were chosen due to the lack of available ranges in the literature. BR: background regimen; ICER: incremental cost-effectiveness ratio; QALY: quality adjusted life-year; TB: tuberculosis

The results of the deterministic sensitivity analysis are presented in Table 5, Table 6, and Fig. 4. Bedaquiline plus BR was cost-saving and more effective than BR alone in the majority of deterministic analyses, with two exceptions. Firstly, where initial care for MDR-TB was managed in an outpatient setting for 100% of cases; in this analysis, bedaquiline plus BR was more costly and more effective than BR alone with an incremental cost per QALY gained of £7,842. Secondly, where the higher mortality rate observed in the bedaquiline arm of the C208 trial was included; in this analysis, bedaquiline plus BR was less costly and less effective than BR alone with an incremental cost per QALY gained of £12,289. Bedaquiline plus BR was cost-saving versus BR alone in cases where the price of bedaquiline was increased by 20%, the cost of BR increased by 20%, where UK (‘real-world’) clinical data were included (based on treatment outcomes for drug-resistant TB in the UK) [40], and when treatment benefit for bedaquiline was restricted to sputum culture conversion (no benefit in reducing the rate of relapse post-culture conversion).

**Table 5:** Sensitivity analysis results (scenario analyses).

Analysis	Treatment	Total costs	Total QALYs gained	Total DALYs lost	Incremental cost per QALY gained	Incremental cost per DALY avoided
Acquisition of resistance and reoccurrence included	Bedaquiline + BR	£2,323,029	100.04	218.47	Dominates (-£7,299)	Dominates (-£1,964)
	BR only	£2,483,322	78.09	300.08		
No reversion events included	Bedaquiline + BR	£2,130,979	107.09	179.34	Dominates (-£9,258)	Dominates (-£2,331)
	BR only	£2,331,006	85.48	265.17		
Trial-based mortality rates included	Bedaquiline + BR	£2,299,677	73.36	297.81	£12,289	£6,112
	BR only	£2,403,442	81.80	280.83		
Real-world UK MDR-TB treatment outcomes included	Bedaquiline + BR	£2,260,967	101.65	196.71	Dominates (-£3,002)	Dominates (-£770)
	BR only	£2,310,047	85.31	260.45		

All costs reported in 2013 values

QALYs: quality adjusted life-years; DALYs: disability adjusted life-years; BR: background regimen; TB: tuberculosis

**Table 6:** Sensitivity analysis results (parameter uncertainty).

Analysis	Range	Treatment	Total costs	Total QALYs gained	Total DALYs lost	Incremental cost per QALY gained	Incremental cost per DALY avoided
Time horizon	2-years	Bedaquiline + BR	£2,016,070	24.98	125.99	Dominates (-£35,174)	Dominates (-£2,079)
		BR only	£2,110,252	22.30	171.29		
Percentage treated in hospital/ community	50% / 50%	Bedaquiline + BR	£1,745,021	105.09	187.80	Dominates (-£1,105)	Dominates (-£271)
		BR only	£1,770,251	81.80	280.83		
	0% / 100%	Bedaquiline + BR	£1,319,647	105.09	187.80	£7,842	£1,963
		BR only	£1,137,061	81.80	280.83		
Cost of BR (intensive and continuous)*	Relative change: +20% (intensive value: 831.24; continuous value: £327.96)	Bedaquiline + BR	£2,204,247	105.09	187.80	Dominates (-£10,234)	Dominates (-£2,561)
		BR only	£2,442,543	81.80	280.83		
	Relative change: -20% (intensive value: £554.16; continuous value: £218.64)	Bedaquiline + BR	£2,136,541	105.09	187.78	Dominates (-£9,783)	Dominates (-£2,449)
		BR only	£2,364,341	81.80	280.83		
Utility weight of active TB, no cure*	Relative change: +20% (Value: 0.82)	Bedaquiline + BR	£2,170,394	107.35	187.80	Dominates (-£10,708)	Dominates (-£2,505)
		BR only	£2,403,442	85.59	280.83		
	Relative change: -20% (Value: 0.54)	Bedaquiline + BR	£2,170,394	102.82	187.80	Dominates (-£9,395)	Dominates (-£2,505)
		BR only	£2,403,442	78.01	280.83		
Disability weight of active TB, no cure*	Relative change: +20% (Value: 0.40)	Bedaquiline + BR	£2,170,394	105.09	189.45	Dominates (-£10,009)	Dominates (-£2,487)
		BR only	£2,403,442	81.80	283.16		
	Relative change: -20% (Value: 0.26)	Bedaquiline + BR	£2,170,394	105.09	186.14	Dominates (-£10,009)	Dominates (-£2,523)
		BR only	£2,403,442	81.80	278.50		
Price of bedaquiline*	Relative change: +20% (Value: 22,560)	Bedaquiline + BR	£2,242,837	105.09	187.80	Dominates (-£6,898)	Dominates (-£1,726)
		BR only	£2,403,442	81.80	280.83		
	Relative change: -20% (Value: 15,040)	Bedaquiline + BR	£2,097,950	105.09	187.80	Dominates (-£13,120)	Dominates (-£3,284)
		Bedaquiline + BR	£2,403,442	81.80	280.83		
Discount rate	0%	Bedaquiline + BR	£2,201,252	121.54	352.60	Dominates (-£9,294)	Dominates (-£1,476)
		BR only	£2,458,920	93.82	527.21		
	5%	Bedaquiline + BR	£2,158,485	99.16	154.99	Dominates (-£10,306)	Dominates (-£2,915)
		BR only	£2,382,100	77.47	231.70		

All costs reported in 2013 values

* Fixed ranges of +/- 20% were chosen due to the lack of available ranges in the literature

QALYs: quality adjusted life-years; DALYs: disability adjusted life-years; BR: background regimen; TB: tuberculosis

## Discussion

The results of this study demonstrate that in the UK, adding bedaquiline to the current standard of care for the treatment of MDR-TB would be cost-effective and cost-saving through a range of bedaquiline cost scenarios. In the UK, the standard threshold under which new treatments are considered to be cost-effective is £20,000 to £30,000 per QALY gained [24]. Typically, medicinal treatments in orphan or ultra-orphan indications, in which MDR-TB falls, are considered cost-ineffective under standard thresholds [22]. This is, in part, due to the unique challenges in demonstrating value for money in indications where commercial returns for investment in research and development are naturally limited by the size of the eligible population, and thus producing higher than average drug costs. As shown in this study, bedaquiline in MDR-TB is a case where the increased cost of treatment is offset by the financial rewards of improved treatment, resulting in an overall cost-saving to the health system, assuming bedaquiline is available at the current US list price of US$30,000 (£18,800). For bedaquiline-eligible patients, bedaquiline plus BR is considered a dominant strategy versus BR alone, and even without having to consider the higher willingness to pay thresholds for orphan drugs, bedaquiline would meet the usual criteria for reimbursement in the UK for use in patients who are eligible and likely to benefit from this intervention. At a willingness-to-pay threshold of £20,000 per QALY, the price of bedaquiline could be set to £55,066 (over 2.9 times the US list price); at willingness-to-pay thresholds of £30,000 per QALY and £50,000, the model demonstrates that the corresponding price thresholds of bedaquiline would be £67,151 (3.6 times the US list price) and £91,322 (4.9 times the US list price).

The cost savings associated with the addition of bedaquiline to the BR were due primarily to a considerable decrease in hospitalization costs resulting from faster sputum culture conversion and a higher proportion of patients achieving sputum culture conversion compared with BR alone. This cost-saving outweighed the increased drug acquisition costs, monitoring, and outpatient costs associated with the use of bedaquiline over a 10-year horizon.

The conclusions of this study are consistent with the conclusions of the WHO exploratory cost-effectiveness analysis of bedaquiline [16], which considered the use of treatment in a range of low to middle-income country settings (Russia, Estonia, Philippines, Peru, Nepal, and China). The time horizon of that study was restricted to the period of the bedaquiline phase II trial, 20 months. In the WHO study, bedaquiline plus BR was considered to be cost-effective versus BR alone, under a wide range of assumptions regarding the translation of trial results to current practice. In addition, the WHO cost-effectiveness model was most sensitive to assumptions on the rate of hospitalization, which was similar to the findings of the current study [16].

Importantly, this study builds on the findings of the WHO evaluation by considering an extended time horizon of 10 years, and using data on sputum culture conversion (an outcome validated by WHO Interim Policy Guidance and an external study [14,41]) as opposed to the WHO definition of treatment success [28], to inform decisions on hospital stay and quality of life. It is important that cost-effectiveness analyses are comprehensive when evaluating the future costs and consequences of treatment. Consequently, it is important that the long-term consequences of failing to induce or maintain culture conversion in patients with MDR-TB are accounted for. The model developed in this study included the simulation of secondary attempts to induce conversion, and the simulated accumulation of health care resources, whilst attempting to care for patients who fail treatment. Further, whilst the WHO definition of treatment success may be appropriate in low-to-middle income settings, where routine screening of sputum culture is not always available, clinical decisions on hospitalization in high-income countries such as the UK are often based on sputum culture results, thus requiring an economic evaluation that modeled treatment benefit in terms of sputum culture conversion.

This study evaluated the use of bedaquiline in a high-income country where access to specialized hospital care and drug treatment for MDR-TB is universal, but also costly. However, as demonstrated in the study sensitivity analysis, bedaquiline remains cost-effective in cases where hospital care is restricted, under the assumption that the same level of patient care is provided from the outpatient setting. These results support the generalizability of our conclusions on the cost-effectiveness of bedaquiline in the UK to other high-income settings where hospital care is not routinely provided to patients with active pulmonary MDR-TB.

The key strengths of this study include its comprehensive health state structure, the use of patient-level data to inform state transitions, and the extent to which the model captures UK and WHO guidelines on treatment strategies for MDR-TB. The health state structure of the model was also verified by a leading UK clinician, who confirmed that the model appropriately captured outcomes in patients with MDR-TB in the UK. In addition, model validation indicated that the model showed good consistency with other previously developed models in this area, and direct cost estimates were within published ranges, which have been estimated at between £50,000 and £70,000 [18,27].

The health state structure of the model consisted of a comprehensive set of states developed to represent the major outcomes observed in the bedaquiline placebo-controlled phase II clinical trial (sputum culture conversion, relapse following culture conversion, loss to follow-up and the use of adjunctive surgery for MDR-TB) and outcomes that are likely to have occurred outside of the trial (end of life care, reoccurrence of TB following conversion and mortality in patients who are lost to follow-up). The data inputs in the model included the analysis of patient-level data from the bedaquiline placebo-controlled phase II clinical trial to estimate the time-dependent rate of sputum culture conversion, which has not been captured in previous economic evaluations. The model also captures the time spent receiving different types of care (inpatient care in negative pressure hospital room, and outpatient, community-based care), and the time spent receiving additional interventions prior to treatment completion as recommended by UK clinical practice guidelines.

The model does not capture the mortality imbalance observed in the placebo-controlled phase II clinical trial in the base case, and instead derives the probability of death from different states described in the literature [30,31]. The key rationale for not incorporating the mortality data from the phase II clinical trial of bedaquiline, was that in this trial no reasons for the imbalance were identified [11,14]. In addition, recent data published based on early access (compassionate use) of bedaquiline suggest, a lower mortality rate than has been observed in clinical trials. In a retrospective cohort study of 35 patients treated with bedaquiline in France, only one patient died (3%), and the death was considered unrelated to TB or TB treatment by the investigator [42]. Similarly, an interim analysis of 91 patients treated with bedaquiline in South Africa reported 3 deaths (3.3%), none of which were considered related to bedaquiline by the investigator [43]. Nevertheless, the excess mortality observed in C208 is captured in sensitivity analyses. Results of this analysis showed that bedaquiline was associated with fewer costs and fewer QALYs compared with BR alone, which led to a positive ICER of £12,289. This was driven by the higher number of deaths in the bedaquiline arm compared with BR only, which led to fewer life years, but also smaller treatment and hospitalisation costs.

As with all decision analytic models, the model presented in this study was subject to a number of assumptions, which in turn may be considered limitations of the analysis. In this model, as is the case with many economic analyses, these assumptions were driven by the absence of data. Specifically, the key limitations in the simulation model were the use of trial data to inform the rate of sputum culture conversion in patients in the UK, the assumed zero cost for patients who are lost to follow-up, and the assumption that patients who occupy the treatment completion state have the same health characteristics of the general population.

The primary source of data in the model was a relatively small phase II placebo-controlled trial [13,15,27]), which enrolled MDR-TB and pre-XDR-TB patients from outside the UK who may not be representative of the MDR-TB population in the UK. Nevertheless, bedaquiline + BR remained cost-saving versus BR alone when UK clinical outcomes were used to estimate rates of sputum culture conversion over time [2].

Data on the outcomes of treatment for MDR-TB in the UK are available and presented in annual TB reports by the Health Protection Agency (HPA) [2]. These data were not used in the model because the composition of drug regimens used in the UK cohort was unknown. This uncertainty is compounded by the observation that 72% of patients treated for MDR-TB in the UK achieved treatment success (WHO definition) outcomes at 24 months [2], compared with 56.1% of patients in the placebo group (BR alone) of the trial (at month 20), and these measures are not directly comparable because the contribution of sputum culture conversion to “success” in the UK definition is not well characterized. The difference in treatment success rate between the UK study and trial sites may be due to several factors, including greater rates of antibacterial resistance in ex-UK sites and the general health status of patients in trial site settings, as well as differences in severity of disease between the general MDR-TB population in the UK and the trial population (all patients in the trial had sputum smear positive TB and over 80% had at least one lung cavity measuring at least 2 centimeters on enrollment). To address this uncertainty, a sensitivity analysis of model inputs was conducted to test for variations in sputum culture conversion. The results of this analysis (Table 5 and Fig. 4) demonstrated that study conclusions were not affected by variations in the rate of sputum culture conversion.

A further limitation in our model is the assumption that patients who were lost to follow-up remained so until death. No costs were applied to this patient group, assuming they do not seek medical intervention. These patients were also assumed to have active TB and experienced the quality of life of patients consistent with active disease. It is possible that a proportion of patients previously lost to follow-up would re-enter care and treatment at a later date. To underscore this point, an audit of TB patients initially classified as lost to follow-up in West Yorkshire in the UK found that 16% of patients later restarted and completed treatment [44]. This limitation is not likely to impact the results of the simulation, given that the rate of loss to follow-up was assumed to be the same between treatment strategies.

The model also assumed that patients who achieved sputum culture conversion did not experience any lasting effects in terms of quality of life or disability, including recurrence of TB. This is likely to be an optimistic assumption, as it has been shown that quality of life remains significantly worse in patients with TB than in the general population, even after they have successfully completed treatment [45]. The impact of previous TB infection on the utility weight of patients who have completed treatment for MDR-TB is currently unknown.

Future enhancements to the simulation model may include the consideration of new and emerging therapies in the treatment of MDR-TB such as delamanid, which has recently been approved for use in MDR-TB in the EU. In addition, the BR treatment duration in the model was 20 months, but there is evidence to suggest that a shorter 9-month BR duration is effective [46]. The effect of adding bedaquiline to a 9-month BR will be studied in Phase 3 clinical trials, and may be considered in future model simulations.

The current model does not explicitly model transmission dynamics of TB. We explored possibilities for attempting to calculate the number of incremental transmitted cases that could be prevented due to the higher and faster sputum culture conversion rates when treated with bedaquiline. A heuristic approach was used to estimate the transmission rate per month, based on the assumption that for each case of TB lasting a year, 0.24 additional secondary cases of TB arise. The calculation is based on the ratio of adult to children TB cases as reported by the TB 2012 annual report (0.24) [2], an established proxy for secondary cases, and the assumption of 1 year (13 months) exposure.

Studies in various settings (US [47,48], Australia [49], Peru [50,51], India [52], and UK [53]) estimate that a typical MDR-TB patient transmits latent infection to between six and twenty contacts per year of infectiousness which, in turn, results in 0.3 to 2.3 (direct estimate) new active cases of MDR-TB each year [48,51,54–56]; therefore the figure derived from the UK proxy data is likely conservative.

Although this analysis is not considered in the base case results presented in the current paper, we find that using this approach, treating a cohort of 20 patients with bedaquiline + BR, compared with BR alone, would result in preventing 0.96 additional MDR-TB cases per year, with a cost saving of £233,048. Including this measure would therefore make the results even more cost-effective than as currently presented. However, the number of secondary TB cases resulting from community transmission in the model should be treated as an estimate only, and more sophisticated disease transmission methods should be used if transmission is to be the primary outcome of interest.

In summary, the results of this study show that treatment with bedaquiline is likely (81% certainty) to produce cost-savings for the UK NHS if sold at the US list price or within +/- 20% of that price, and lead to improvements in the average quality of life of patients with MDR-TB. Bedaquiline, when added to current standard of care for treating MDR-TB, should be considered the dominant treatment strategy (less costly and more effective) compared with the standard of care alone. These results support the use of bedaquiline in eligible patients.

## Supporting Information

S1 Appendix Explanation of the model pathway. (DOCX)

S2 Appendix Conversion of hazard functions to probabilities. (DOCX)

S1 Fig Trial-based estimates of culture conversion for bedaquiline + BR versus BR alone (log-normal). BR: background regimen
(TIF)

S1 Table Distribution of health outcomes at 1 year. (DOCX)

S2 Table Probabilistic distributions, parameters and definitions as used in the PSA. (DOCX)

## References

[1] WHO. Tuberculosis Fact Sheet Number 104. October 2014. Available: http://www.who.int/mediacentre/factsheets/fs104/en/index.html. Accessed 2015 Feb 12.

[2] HPA. Tuberculosis in the UK: 2013 report. January 2014. Available: https://www.gov.uk/government/uploads/system/uploads/attachment_data/file/325632/TB_in_the_UK.pdf. Accessed 2015 Feb 12.

[3] ECDC. Tuberculosis surveillance and monitoring in Europe. 2013. Available: http://www.ecdc.europa.eu/en/publications/Publications/Tuberculosis-surveillance-monitoring-2013.pdf. Accessed 2015 Feb 12.

[4] British Thoracic Society. Chemotherapy and management of tuberculosis in the United Kingdom: recommendations 1998. Joint Tuberculosis Committee of the British Thoracic Society. Thorax. 1998; 53: 536–548. 9797751 PMC1745276

[5] Curry International TB Center. CITC (Curry International Tuberculosis Center) and TBCB (TB control branch) of California department, Drug-resistant tuberculosis: a survival guide for clinicians. 2008.

[6] US CDC. Multidrug-Resistant Tuberculosis (MDR TB) and Extensively-Drug Resistant (XDR) TB. 2007.

[7] WHO. Global Tuberculosis Report 2013. 2013. Available: http://apps.who.int/iris/bitstream/10665/91355/1/9789241564656_eng.pdf. Accessed 2015 Feb 12.

[8] Brigden G, Nyang'wa BT, du CP, Varaine F, Hughes J, Rich M, et al Principles for designing future regimens for multidrug-resistant tuberculosis. Bull World Health Organ. 2014; 92: 68–74. 10.2471/BLT.13.122028 24391302 PMC3865549

[9] WHO. Stop TB Partnership. The Global Plan to stop TB 2011–2015. Transforming the fight towards elimination of tuberculosis. 2011. Available: http://www.stoptb.org/assets/documents/global/plan/tb_globalplantostoptb2011-2015.pdf. Accessed 2015 Feb 12.

[10] FDA. FDA News Release: Approval of bedaquiline as part of a combination therapy to treat adults with multi-drug resistant pulmonary tuberculosis. 31 December 2012.

[11] European Medicines Agency. EMA/CHMP/771324/2013—EMA Committee for Medicinal Products for Human Use (CHMP), Summary of opinion (initial authorisation)—Sirturo (bedaquiline). 19 December 2013.

[12] Janssen. Sirturo: highlights of US Prescribing Information. 2013. Available: http://www.accessdata.fda.gov/drugsatfda_docs/label/2012/204384s000lbl.pdf. Accessed 12 February 2015.

[13] FDA. Anti-Infective Drugs Advisory Committee Meeting: TMC207 (Bedaquiline). December 2012. Available: http://www.fda.gov/downloads/AdvisoryCommittees/CommitteesMeetingMaterials/Drugs/Anti-InfectiveDrugsAdvisoryCommittee/UCM329260.pdf. Accessed 2014 Sep 1.

[14] WHO. The use of bedaquiline in the treatment of multidrug-resistant tuberculosis. Interim policy guidance. 2013. Available: http://apps.who.int/iris/bitstream/10665/84879/1/9789241505482_eng.pdf. Accessed 2015 Feb 12. 23967502

[15] Diacon AH, Pym A, Grobusch MP, de los Rios JM, Gotuzzo E, Vasilyeva I, et al Multidrug-resistant tuberculosis and culture conversion with bedaquiline. N Engl J Med. 2014; 371: 723–732. 10.1056/NEJMoa1313865 25140958

[16] WHO, Vassell A. Cost-effectiveness of introducing bedaquiline in MDR-TB regimens—an exploratory analysis. 26 January 2013. Available: http://who.int/tb/challenges/mdr/CEA_bdqreport_final.pdf. Accessed 2015 Feb 12.

[17] White VL, Moore-Gillon J. Resource implications of patients with multidrug resistant tuberculosis. Thorax. 2000; 55: 962–963. 11050268 10.1136/thorax.55.11.962 PMC1745633

[18] Diel R, Vandeputte J, De Vries G, Stillo J, Wanlin M, Nienhaus A. Costs of tuberculosis disease in the EU—a systematic analysis and cost calculation. Eur Respir J. 2013. 10.1183/09031936.00079413 23949960

[19] EMA. Orphan designation. 2014. Available: http://www.ema.europa.eu/ema/index.jsp?curl=pages/regulation/general/general_content_000029.jsp. Accessed 2015 Feb 15.

[20] European Commission. Community register of medicinal products for human use: bedaquiline product information. 2015. Available: http://ec.europa.eu/health/documents/community-register/html/h901.htm. Accessed 2015 Feb 15.

[21] Rawlins MD, Culyer AJ. National Institute for Clinical Excellence and its value judgments. BMJ. 2004; 329: 224–227. 15271836 10.1136/bmj.329.7459.224 PMC487742

[22] Drummond MF, Wilson DA, Kanavos P, Ubel P, Rovira J. Assessing the economic challenges posed by orphan drugs. Int J Technol Assess Health Care. 2007; 23: 36–42. 17234015 10.1017/S0266462307051550

[23] Siebert U, Alagoz O, Bayoumi AM, Jahn B, Owens DK, Cohen DJ, et al State-transition modeling: a report of the ISPOR-SMDM Modeling Good Research Practices Task Force—3. Value Health. 2012; 15: 812–820. 10.1016/j.jval.2012.06.014 22999130

[24] NICE. Measuring effectiveness and cost effectiveness: the QALY. April 2010. Available: http://www.nice.org.uk/newsroom/features/measuringeffectivenessandcosteffectivenesstheqaly.jsp. Accessed 2014 Sep 1.

[25] Murray CJL. Quantifying the burden of disease: the technical basis for disability-adjusted life years. Bulletin of the World Health Organization. 1994; 72: 429–445. 8062401 PMC2486718

[26] NICE. Methods for the development of NICE public health guidance (second edition). 2009 London, UK.

[27] WHO. Guidelines for the programmatic management of drug-resistant tuberculosis. 2008. Available: http://whqlibdoc.who.int/publications/2008/9789241547581_eng.pdf. Accessed 2015 Feb 12.

[28] WHO. Definitions and reporting framework for tuberculosis—2013 revision. Available: http://apps.who.int/iris/bitstream/10665/79199/1/9789241505345_eng.pdf. Accessed 2015 Feb 12.

[29] Franke MF, Appleton SC, Bayona J, Arteaga F, Palacios E, Llaro K, et al Risk factors and mortality associated with default from multidrug-resistant tuberculosis treatment. Clin Infect Dis. 2008; 46: 1844–1851. 10.1086/588292 18462099 PMC2577177

[30] Liu CH, Li L, Chen Z, Wang Q, Hu YL, Zhu B, et al Characteristics and treatment outcomes of patients with MDR and XDR tuberculosis in a TB referral hospital in Beijing: a 13-year experience. PLoS One. 2011; 6: e19399 10.1371/journal.pone.0019399 21559362 PMC3084844

[31] Tiemersma EW, van der Werf MJ, Borgdorff MW, Williams BG, Nagelkerke NJ. Natural history of tuberculosis: duration and fatality of untreated pulmonary tuberculosis in HIV negative patients: a systematic review. PLoS One. 2011; 6: e17601 10.1371/journal.pone.0017601 21483732 PMC3070694

[32] Lunn D, Thomas A, Best N, Spiegelhalter D. WinBUGS—A Bayesian modelling framework: Concepts, structure, and extensibility. Statistics and Computing. 2000; 10: 325–337.

[33] Department of Health. British National Formulary. 2013. Available: http://www.bnf.org/bnf/index.htm. Accessed 2014 Sep 1.

[34] Department of Health. NHS Reference Costs 2011–2012. 2012. Available: https://www.gov.uk/government/uploads/system/uploads/attachment_data/file/213060/2011-12-reference-costs-publication.pdf. Accessed 2015 Feb 12.

[35] NICE. CG117: Tuberculosis: Clinical diagnosis and management of tuberculosis, and measures for its prevention and control. 2011. Available: http://www.nice.org.uk/guidance/cg117/resources/cg117-tuberculosis-full-guideline3. Accessed 2015 Feb 12.

[36] Jit M, Stagg HR, Aldridge RW, White PJ, Abubakar I. Dedicated outreach service for hard to reach patients with tuberculosis in London: observational study and economic evaluation. BMJ. 2011; 343: d5376 10.1136/bmj.d5376 22067473 PMC3273731

[37] Kind P, Hardman G, Macran S. UK population norms for EQ-5D. November 1999. Available: https://www.york.ac.uk/media/che/documents/papers/discussionpapers/CHE%20Discussion%20Paper%20172.pdf. Accessed 2015 Feb 12.

[38] Salomon JA, Vos T, Hogan DR, Gagnon M, Naghavi M, Mokdad A, et al Common values in assessing health outcomes from disease and injury: disability weights measurement study for the Global Burden of Disease Study 2010. Lancet. 2012; 380: 2129–2143. 10.1016/S0140-6736(12)61680-8 23245605 PMC10782811

[39] ONS. Office for National Statistics Life Tables 2009–2011—England and Wales. 2013. Available: http://www.ons.gov.uk/ons/rel/lifetables/interim-life-tables/2009-2011/rft-england-and-wales.xls. Accessed 2014 Sep 1.

[40] Health Protection Agency. Tuberculosis in the UK: Annual report on tuberculosis surveillance in the UK, 2013. Available: https://www.gov.uk/government/uploads/system/uploads/attachment_data/file/325632/TB_in_the_UK.pdf. Accessed 2015 Feb 12.

[41] Kurbatova E, Smith S, Cegielski J. Evaluation of sputum culture conversion as a surrogate marker of treatment outcome in patients with multidrug-resistant tuberculosis. 2013. CDC, Atlanta, GA, United States.

[42] Guglielmetti L, Le DD, Jachym M, Henry B, Martin D, Caumes E, et al. Compassionate Use of Bedaquiline for the Treatment of Multidrug-Resistant and Extensively Drug-Resistant Tuberculosis: Interim Analysis of a French Cohort. Clin Infect Dis. 2014; ciu786 [pii];10.1093/cid/ciu786. 10.1093/cid/ciu786 25320286

[43] Ndjeka N, Conradie F, Hughes J, Schnipel K, Cox H, Bantubani N, et al Safe and effective bedaquiline treatment of drug-resistant tuberculosis (DR-TB) within the National Bedaquiline Clinical Access Programme in South Africa. S Afr Med J. 2014; 104(3):164–166 24897814 10.7196/samj.7263

[44] Day M, Middlemiss A, Thorpe J, Okereke E. What really happens to tuberculosis patients classified as lost to follow-up in West Yorkshire? Euro Surveill. 2012; 17. 23040967

[45] Guo N, Marra F, Marra CA. Measuring health-related quality of life in tuberculosis: a systematic review. Health Qual Life Outcomes. 2009; 7:14 10.1186/1477-7525-7-14 19224645 PMC2651863

[46] Van Deun A, Maug AK, Salim MA, Das PK, Sarker MR, Daru P, et al Short, highly effective, and inexpensive standardized treatment of multidrug-resistant tuberculosis. Am J Respir Crit Care Med. 2010; 182: 684–692. 10.1164/rccm.201001-0077OC 20442432

[47] Blower SM, Chou T. Modeling the emergence of the 'hot zones': tuberculosis and the amplification dynamics of drug resistance. Nat Med. 2004; 10: 1111–1116. 15378053 10.1038/nm1102

[48] Murphy BM, Singer BH, Anderson S, Kirschner D. Comparing epidemic tuberculosis in demographically distinct heterogeneous populations. Math Biosci. 2002; 180: 161–185. 12387922 10.1016/s0025-5564(02)00133-5

[49] Denholm JT, Leslie DE, Jenkin GA, Darby J, Johnson PD, Graham SM, et al Long-term follow-up of contacts exposed to multidrug-resistant tuberculosis in Victoria, Australia, 1995–2010. Int J Tuberc Lung Dis. 2012; 16: 1320–1325. 10.5588/ijtld.12.0092 22863690

[50] Grandjean L, Crossa A, Gilman RH, Herrera C, Bonilla C, Jave O, et al Tuberculosis in household contacts of multidrug-resistant tuberculosis patients. Int J Tuberc Lung Dis. 2011; 15:1164–9. 10.5588/ijtld.11.0030 21943839

[51] Resch SC, Salomon JA, Murray M, Weinstein MC. Cost-effectiveness of treating multidrug-resistant tuberculosis. PLoS Med. 2006; 3: e241 16796403 10.1371/journal.pmed.0030241 PMC1483913

[52] Singla N, Singla R, Jain G, Habib L, Behera D. Tuberculosis among household contacts of multidrug-resistant tuberculosis patients in Delhi, India. Int J Tuberc Lung Dis. 2011; 15:1326–1330. 10.5588/ijtld.10.0564 22283889

[53] Neely F, Maguire H, Le BF, Davies A, Gelb D, Yates S. High rate of transmission among contacts in large London outbreak of isoniazid mono-resistant tuberculosis. J Public Health (Oxf). 2010; 32: 44–51. 10.1093/pubmed/fdp056 19542269

[54] Luciani F, Sisson SA, Jiang H, Francis AR, Tanaka MM. The epidemiological fitness cost of drug resistance in Mycobacterium tuberculosis. Proc Natl Acad Sci U S A. 2009; 106: 14711–14715. 10.1073/pnas.0902437106 19706556 PMC2732896

[55] Cohen T, Murray M. Modeling epidemics of multidrug-resistant M. tuberculosis of heterogeneous fitness. Nat Med. 2004; 10: 1117–1121. 15378056 10.1038/nm1110 PMC2652755

[56] Liao CM, Cheng YH, Lin YJ, Hsieh NH, Huang TL, Chio CP, et al A probabilistic transmission and population dynamic model to assess tuberculosis infection risk. Risk Anal. 2012; 32: 1420–1432. 10.1111/j.1539-6924.2011.01750.x 22211354

