# Supplementary material for: Cost-Effectiveness of Adding Bedaquiline to Drug Regimens for the Treatment of Multidrug-Resistant Tuberculosis in the UK
Source: PLoS One. 2015 Mar 20;10(3):e0120763. doi: 10.1371/journal.pone.0120763 (PMC4368676; doi:10.1371/journal.pone.0120763)
Supplement: S1 Appendix — (DOCX) [file pone.0120763.s001.docx]

**Technical appendix S1: Explanation of the model pathway**

At the start of the time horizon, the simulation cohort entered the model in the ‘active MDR-TB’ state. Patients occupying this state received a treatment programme for MDR-TB, and were assumed to have not yet achieved sputum culture conversion.

Over the course of successive monthly transition cycles, patients in the active TB states were able to:

- Retain active TB status
- Achieve sputum culture conversion and transition to the ‘sputum converted MDR-TB‘ state
- Receive adjunctive lung resection surgery and transition to the ‘surgery (short-term outcomes)’ state
- Transfer out or default from the programme and transition to the ‘lost to follow-up, MDR-TB‘ state
- Die, transitioning to the ‘death‘ state

Patients who transited to the surgery and lost to follow-up states were assumed to occupy these states until death. The surgery health states were further defined in terms of short-term and long-term outcomes. The initial cost of surgery and the risk of mortality attributed to the operative period (surgery plus 28 days post-surgery) were captured in the short-term surgery health state. The downstream impact of surgery on outcomes (i.e. cure) were captured in the long-term surgery state.

In practice, the majority of patients who are lost to follow-up remain lost to follow up until death,[1] with some patients developing a higher level of resistance as a result of absent or inadequate treatment.[2] To reflect this, the cost and utility weights applied to these health states were adjusted to reflect the potential downstream consequences of treatment in these patients.

Patients who achieved sputum culture conversion benefited from an improvement in QoL, which increased for each cycle of sustained conversion until reaching the QoL equivalent to that of the general population. For those who achieved sputum culture conversion, follow-on events simulated during each cycle in the model included:

- Retain sputum culture conversion
- Transfer out or default from the programme and transition to the ‘lost to follow-up, MDR-TB‘ state
- Relapse having experienced a negative sputum culture conversion and transition to the ‘active secondary MDR-TB‘ state
- Complete treatment having successful retained sputum culture conversion for the duration of the post-cure treatment phase
- Die and transition to the ‘Death‘ state

In the model, relapse events were defined as the occurrence of a positive sputum culture in patients who previously achieved negative sputum cultures during the post-conversion treatment phase. These events were captured in the model as data from stage 2 of the C208 study showed that of the 38 patients randomised to the placebo arm of the study who had a negative sputum culture status at week 24 (N=66), 13 (N=24) had reverted back to a positive sputum culture by study endpoint (week 120). Further, data from the C208 study demonstrated that the Sirturo^®^ group experienced a lower rate of relapse between week 24 and endpoint than the group randomised to placebo.

For patients who completed treatment having successfully retained sputum culture conversion for the duration of the post-cure treatment phase, follow-on events included:

- Remain TB-free (no transition)
- Die and transition to the ‘Death‘ state

In the model, patients who developed secondary active MDR-TB experienced the same core outcomes as patients who have active MDR-TB (culture conversion, lost to follow-up, surgery). There were however, differences between the pathways of care for MDR-TB and secondary MDR-TB.

Throughout the simulation, patients with secondary MDR-TB were unable to develop further resistance or acquire new infection post-treatment completion.

**References**

1. Franke MF, Appleton SC, Bayona J, Arteaga F, Palacios E, Llaro K, Shin SS, Becerra MC, Murray MB, Mitnick CD (2008) Risk factors and mortality associated with default from multidrug-resistant tuberculosis treatment. Clin Infect Dis 46: 1844-1851.

2. Wilton P, Smith RD, Coast J, Millar M, Karcher A (2001) Directly observed treatment for multidrug-resistant tuberculosis: an economic evaluation in the United States of America and South Africa. Int J Tuberc Lung Dis 5: 1137-1142.
