# Supplementary material for: Cost-Effectiveness of Adding Bedaquiline to Drug Regimens for the Treatment of Multidrug-Resistant Tuberculosis in the UK
Source: PLoS One. 2015 Mar 20;10(3):e0120763. doi: 10.1371/journal.pone.0120763 (PMC4368676; doi:10.1371/journal.pone.0120763)
Supplement: S2 Appendix — (DOCX) [file pone.0120763.s002.docx]

**Technical appendix S2: Conversion of hazard functions to probabilities**

The survival curve for the complete time horizon of the model (20 years) was calculated using the following steps:

1. Cumulative survival probabilities calculated for cycle 1 (day 0 – 28), and cycle 2 (day 28 – 56) using the week 0 – 8 survival curve and equation (1)
2. Cumulative survival probability for cycle 3 (day 56-84) calculated as the cumulative survival at cycle 2 (calculated in previous step) multiplied by cumulative survival probability for the first 4-weeks of the week 8 – 24 analysis
3. Step ii repeated up to week 24
4. Cumulative survival probability for week 24 – 28 calculated as the survival at week 20 – 24 from step iii, multiplied by the cumulative survival probability for the first 4-weeks of the week 24+ analysis
5. Step iv repeated for duration of time horizon

The cumulative survival probability equation is defined as follows:

$$s\left( t \right)= 1-\theta\left( \frac{\ln\left( t \right)-\beta}{\sigma} \right)$$

(1)

Where, θ is the standard cumulative normal distribution, β is the scale parameter and σ is the shape parameter.

For each segment (0 – 8 weeks, 8– 24 weeks, 24+ weeks), the probability of transitioning from active TB to sputum culture converted TB was estimated using the following formula:

$$Tp=1-\frac{s(t)}{s(t-u)}$$

(2)

Where, Tp is the transition probability, s(t) the survival function at time t, and u is the cycle length (1 month).
