# Supplementary figures and images for: Cost-Effectiveness of Adding Bedaquiline to Drug Regimens for the Treatment of Multidrug-Resistant Tuberculosis in the UK
Source: PLoS One. 2015 Mar 20;10(3):e0120763. doi: 10.1371/journal.pone.0120763 (PMC4368676; doi:10.1371/journal.pone.0120763)

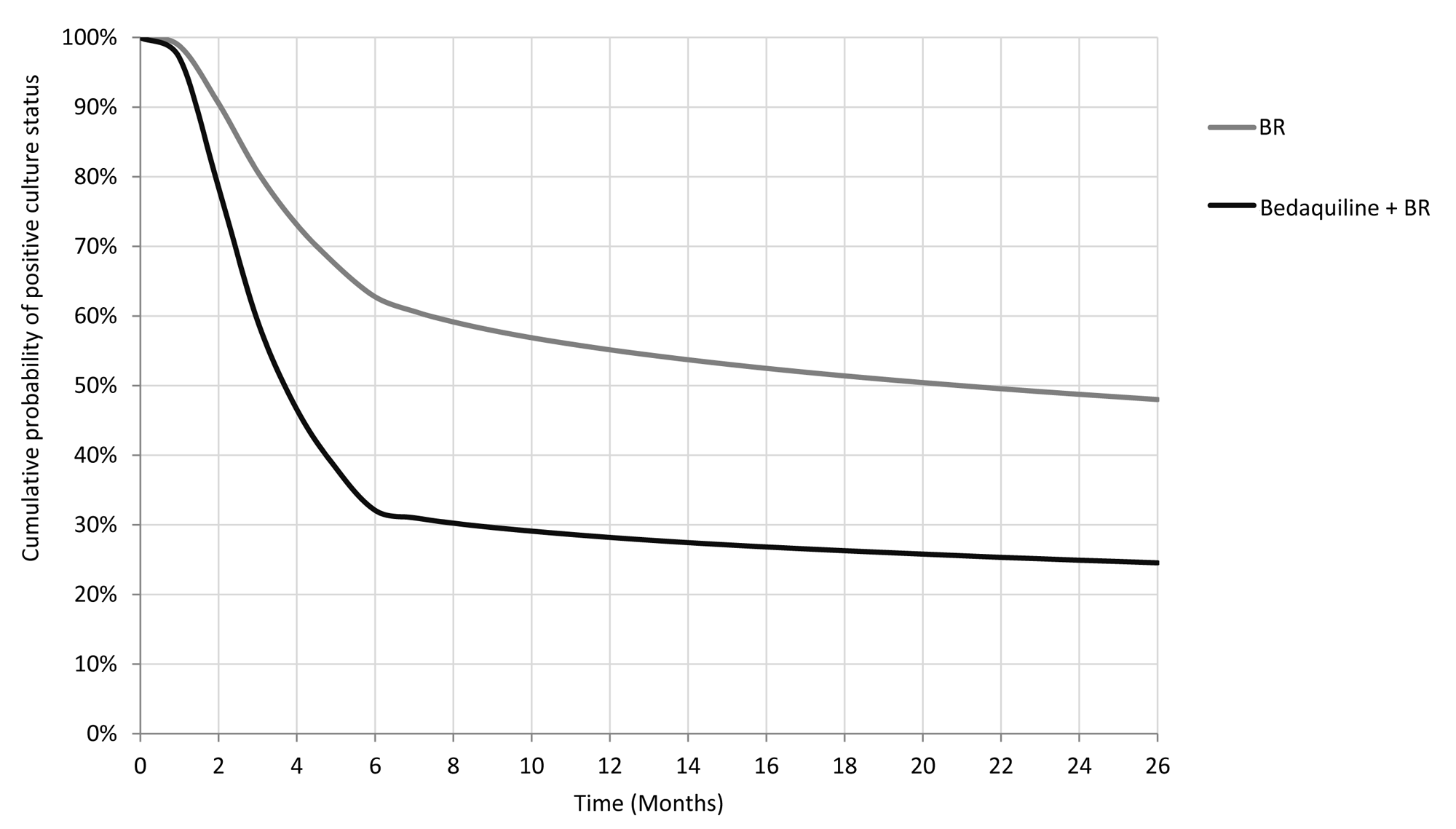

Supplement: S1 Fig — BR: background regimen (TIF) [file pone.0120763.s003.tif]
