# Supplementary material for: Cost-Effectiveness of Adding Bedaquiline to Drug Regimens for the Treatment of Multidrug-Resistant Tuberculosis in the UK
Source: PLoS One. 2015 Mar 20;10(3):e0120763. doi: 10.1371/journal.pone.0120763 (PMC4368676; doi:10.1371/journal.pone.0120763)
Supplement: S1 Table — (DOCX) [file pone.0120763.s004.docx]

Table S1: Distribution of health outcomes at 1 year

| Analysis | Regimen | Outcome |
| --- | --- | --- |
| Percentage of patients with successful outcomes | Bedaquiline + BR | 62.72% |
|  | BR only | 43.52% |
| Total LYs | Bedaquiline + BR | 125.06 |
|  | BR only | 102.23 |
| Number of patients with active TB | Bedaquiline + BR | 4.2 |
|  | BR only | 8.2 |
| Number of culture-converted patients still receiving treatment | Bedaquiline + BR | 12.3 |
|  | BR only | 7.4 |
| Number of patients with no TB | Bedaquiline + BR | 0 |
|  | BR only | 0 |
| Number of patients lost to follow-up, receiving end-of-life care, or dead | Bedaquiline + BR | 3.9 |
|  | BR only | 4.8 |

BR: Background regimen; LYs: life years; TB: tuberculosis
