# Supplementary material for: Cost-Effectiveness of Adding Bedaquiline to Drug Regimens for the Treatment of Multidrug-Resistant Tuberculosis in the UK
Source: PLoS One. 2015 Mar 20;10(3):e0120763. doi: 10.1371/journal.pone.0120763 (PMC4368676; doi:10.1371/journal.pone.0120763)
Supplement: S2 Table — (DOCX) [file pone.0120763.s005.docx]

**Table S2: Probabilistic distributions, parameters and definitions as used in the PSA**

| Parameter | Distribution | Mean | Standard error | Alpha parameters (base case analysis) | Beta parameters (base case analysis) |
| --- | --- | --- | --- | --- | --- |
| Lambda (scale parameter) for cure rates, 0-8 weeks, BR only | Multivariate log-normal^1^ | 5.20 | 0.21 | - | - |
| Treatment parameter (only used in PSA) | Multivariate log-normal^1^ | 0.23 | 0.19 | - | - |
| Gamma (shape parameter) for cure rates, 0-8 weeks, BR only | Multivariate log-normal^1^ | 0.64 | 0.11 | - | - |
| Lambda (scale parameter) for cure rates, 8-24 weeks, BR only | Multivariate log-normal^1^ | 6.09 | 0.40 | - | - |
| Treatment parameter (only used in PSA) | Multivariate log-normal^1^ | 0.39 | 0.48 | - | - |
| Gamma (shape parameter) for cure rates, 8-24 weeks, BR only | Multivariate log-normal^1^ | 1.72 | 0.27 | - | - |
| Lambda (scale parameter) for cure rates, 24+ weeks, BR only | Multivariate log-normal^1^ | 8.65 | 1.09 | - | - |
| Treatment parameter (only used in PSA) | Multivariate log-normal^1^ | 1.24 | 1.63 | - | - |
| Gamma (shape parameter) for cure rates, 24+ weeks, BR only | Multivariate log-normal^1^ | 2.45 | 0.82 | - | - |
| BR exponential percentage | Multivariate log-normal^1^ | 0.75 | 81.00* | - | - |
| Hazard ratio on sputum culture conversion for bedaquiline | Log-normal^2^ | 1.87 | 0.57 | - | - |
| Hazard ratio on relapse for bedaquiline | Log-normal^2^ | 0.39 | 0.25 | - | - |
| Hazard ratio of patients on subsequent MDR-TB treatment compared with initial MDR treatment | Log-normal^2^ | 0.94 | 0.17 | - | - |
| Probability of lost to follow up - initial MDR-TB treatment | Beta^3^ | 0.00 | 0.00 | 6.04 | 1560.46 |
| Probability of lost to follow up - subsequent MDR-TB treatment | Beta^3^ | 0.00 | 0.00 | 6.04 | 1560.46 |
| Probability of surgery per month, >24 weeks - initial MDR-TB treatment | Beta^3^ | 0.00 | 0.00 | 6.93 | 1834.51 |
| Probability of surgery per month, >24 weeks - subsequent MDR-TB treatment | Beta^3^ | 0.01 | 0.00 | 6.93 | 1834.51 |
| % of patients with successful surgery - initial MDR-TB treatment | Beta^3^ | 0.83 | 0.03 | 172.64 | 32.88 |
| % of patients with successful surgery - subsequent MDR-TB treatment | Beta^3^ | 0.80 | 0.03 | 172.64 | 32.88 |
| Mortality rate associated with surgical procedure | Beta^3^ | 0.04 | 0.02 | 6.00 | 114.00 |
| Probability of death following lost to follow up - initial MDR-TB treatment | Beta^3^ | 0.04 | 0.10 | 0.38 | 5.12 |
| Probability of death following lost to follow up - subsequent MDR-TB treatment | Beta^3^ | 0.04 | 0.10 | 0.38 | 5.12 |
| Probability of death for end of life care | Beta^3^ | 0.05 | 0.01 | 23.65 | 431.84 |
| % of patients who undergo surgery with successful outcome | Beta^3^ | 0.82 | 0.03 | 172.64 | 32.88 |
| % of patients who undergo surgery with unsuccessful outcome | Beta^3^ | 0.18 |  | - | - |
| Mean total cost (negative pressure room) | Log-normal^2^ | 19012.56 | 7576.56 | - | - |
| Extra bed days | Log-normal^2^ | 284.95 | 86.45 | - | - |
| Cost of BR | Beta^3^ | Unique to each treatment | 81.00 | - | - |
| Cost of surgery - short term | Gamma^4^ | 2840.42 | 3559.00 | 3.55 | 1888.58 |
| Utility weight of patient with MDR-TB, not cured, on treatment | Beta^3^ | 0.63 | 0.14 | 7.32 | 3.44 |
| Utility weight of patient with MDR-TB, cured, on treatment (first year) | Beta^3^ | 0.86 | 0.04 | 81.12 | 21.56 |
| Utility weight of patient with MDR-TB, cured, on treatment (subsequent year) | Beta^3^ | 0.84 | 0.04 | 77.10 | 18.09 |
| Utility weight of MDR-TB patient undergoing surgery | Log-normal^2^ (on disutilities | 0.84 | - | - | - |
| Utility weight of MDR-TB patient post-surgery | Log-normal^2^ (on disutilities) | 0.98 | - | - | - |
| Utility weight of MDR-TB patient lost to follow-up | Log-normal^2^ (on disutilities) | 0.79 | - | - | - |
| Disability weight of patient with MDR-TB, not cured, on treatment | Beta^3^ | 0.44 | 0.06 | 21.41 | 43.12 |
| Disability weight of patient with MDR-TB, cured, on treatment | Beta^3^ | 0.12 | 0.06 | 6.62 | 33.28 |
| Disability weight of MDR-TB patient undergoing surgery | Beta^3^ | 0.32 | 0.06 | 21.41 | 43.12 |
| Disability weight of MDR-TB patient post-surgery | Beta^3^ | 0.08 | 0.06 | 0.74 | 13.12 |
| Disability weight of MDR-TB patient lost to follow-up | Beta^3^ | 0.33 | 0.06 | 21.41 | 43.12 |
| General population disutility weights | Log-normal^2^ (on disutilities) | Unique to each age group | - | - | - |

*Sample size

^1^Multivariate lognormal distribution: A log-normal distribution is bounded between 0 and infinity, and skewed. A multivariate distribution was used to take into account the correlation between the shape, scale, and treatment parameter outputted by the patient level analysis

^2^Lognormal distribution: Bounded between 0 and infinity, and skewed

^3^Beta distribution: Bounded between 0 and 1

^4^Gamma distribution: Bounded between 0 and infinity, and skewed

BR: Background Regimen; MDR-TB: Multi-Drug-Resistant Tuberculosis; PSA: Probabilistic Sensitivity Analysis; TB: Tuberculosis
